# Differences in DNA Methylation in Genes Involved in Vitamin D Metabolism Are Related to Insulin Requirement in Pregnant Women with Gestational Diabetes Mellitus

**DOI:** 10.3390/ijms251910576

**Published:** 2024-10-01

**Authors:** Nerea Peña-Montero, Teresa María Linares-Pineda, Andrea Fernández-Valero, Fuensanta Lima-Rubio, Ana María Fernández-Ramos, Carolina Gutiérrez-Repiso, María Suárez-Arana, María José Picón-César, María Molina-Vega, Sonsoles Morcillo

**Affiliations:** 1 Department of Endocrinology and Nutrition, Virgen de la Victoria University Hospital, 29010 Málaga, Spain; nereamontero4@gmail.com (N.P.-M.); teresamaria712@gmail.com (T.M.L.-P.); andreafeva@gmail.com (A.F.-V.); santi.lima@hotmail.com (F.L.-R.); carogure@hotmail.com (C.G.-R.); mjpiconcesar@gmail.com (M.J.P.-C.); molinavegamaria@gmail.com (M.M.-V.); 2 CIBER Pathophysiology of Obesity and Nutrition—CIBERON, 28029 Madrid, Spain; 3 Biomedical Research Institute—IBIMA, 29590 Málaga, Spain; 4 Department of Clinical Analysis, Virgen de la Victoria University Hospital, 29010 Málaga, Spain; amfrlab@gmail.com; 5 Department of Obstetrics and Gynecology, Regional University Hospital, 29011 Málaga, Spain; dramariasuarez@gmail.com

**Keywords:** gestational diabetes mellitus, vitamin D, insulin, metabolism, DNA methylation

## Abstract

In a previous study performed by our group, pregnant women with Gestational Diabetes (GDM) showed higher vitamin D (VitD) levels in the last trimester, particularly in those requiring insulin. This phenomenon was not linked to factors like season or supplementation. This study aimed to investigate if insulin treatment in GDM affects DNA methylation in VitD metabolism genes. Thirty-two pregnant women were selected, half of whom had GDM, and were divided into insulin-treated and lifestyle groups. The DNA methylation levels in CpGs from 47 VitD metabolism-related genes were analyzed at the diagnostic visit (24–28 weeks) and before delivery. At week 36–38 of pregnancy, twenty-six CpG sites were differentially methylated (DMPs) in the insulin-treated group compared with the control group and the lifestyle group. Twenty-two of these DMPs were not different at the diagnostic visit. Six CpGs (cg18276810 (CTNNB1), cg03919554 (FGFR3), cg03984919 (NCOA1), cg19218509 (ASIP), cg09922639 (SMAD3), and cg25356935 (PDZD3)) showed significant correlations with VitD levels, not only before childbirth, but also in the postpartum period and at one year later. This suggests that insulin treatment in GDM could influence DNA methylation in genes involved in vitamin D metabolism, affecting VitD levels during and after pregnancy. Further research is warranted to elucidate these findings’ clinical implications.

## 1. Introduction

Vitamin D is now recognized not only as a vitamin but also as a hormone and has received high attention from researchers during the last century [1]. In addition to its known role in calcium and bone homeostasis, potential extra-skeletal effects of vitamin D have been suggested, based on the expression of the vitamin D receptor (VDR) in tissues not related to calcium homeostasis such as the pancreas, pituitary, breast, colon and prostate cancer cells, and immune cells [2]. Although a low vitamin D status has been related to a higher risk of many diseases, such as type 2 diabetes mellitus (T2DM), cardiovascular disease, or cancer, among others, clinical trials have not shown the efficacy of vitamin D in preventing them [1].

Vitamin D has also been related to Gestational Diabetes Mellitus (GDM). Previous studies have reported a higher risk of developing GDM among women with lower levels of vitamin D [3,4]. However, other studies did not find vitamin D status and GDM risk to be related [5,6] and even Yong et al. observed a higher risk of developing GDM in women with high levels of vitamin D during early pregnancy [7]. In our study population, women with GDM requiring insulin had higher levels of vitamin D in comparison with those not requiring insulin and healthy controls, both at postpartum and 1 year after pregnancy [8]. We hypothesized that epigenetic mechanisms could have a role in vitamin D regulation. 

Epigenetic mechanisms can impact the action of drugs [9]. Some studies, although still limited, have described alterations produced by glucose-lowering agents over the epigenome in human tissues and cells [10,11,12,13]. Moreover, insulin treatment has been shown to regulate the gene expression of genes related to lipogenesis and cell proliferation through DNA methylation [14,15]. 

The aim of this study is to test whether DNA methylation levels in genes involved in vitamin D metabolism differ in the insulin-treated group versus the lifestyle group and the control group throughout pregnancy, in order to test our hypothesis that insulin therapy could be favoring higher levels of vitamin D through epigenetic mechanisms in pregnant women with GDM.

## 2. Results

### 2.1. Characteristics of the Study Subjects across Different Points

Characteristics of the participants and a comparison between groups (control, lifestyle, and insulin) are shown in Table 1. At the diagnostic visit (baseline), age, fasting glucose levels, HOMA-IR, and HbA1c were statistically different between groups; meanwhile, levels of vitamin D were similar. At the antepartum visit, vitamin D levels, HbA1c, and HOMA-IR were statistically significant. Meanwhile, at the postpartum visit, only BMI and vitamin D levels were different between groups. 

### 2.2. DNA Methylation of Genes Involved in Vitamin D Metabolism Pathways

We conducted a search through the KEGG and STRING databases to identify genes that could be related to vitamin D metabolism. This search returned 47 genes involved in pathways associated with vitamin D metabolism (Supplementary Table S1). We extracted the beta values of the CpG sites from these 47 genes included in the EPIC bead cheap array, carried out in our population study (n = 32). A total of 1373 CpG sites were identified in these genes. 

We focused our analysis to identify those CpG sites that were different at the end of pregnancy in the insulin-treated group compared with the other groups (control and lifestyle) but were similar at the diagnostic visit, indicating a change during the last trimester. We observed 76 CpG sites differentially methylated (DMPs) between groups at the antenatal visit, and 26 of them were statistically different in the insulin-treated group in comparison to the control group and the lifestyle group. Next, we tested which of these 26 DMPs were not different at the diagnostic visit. Finally, a total of 22 CpG sites were selected (Table 2). These CpG sites were annotated to 18 coding genes. 

**Figure 1:**
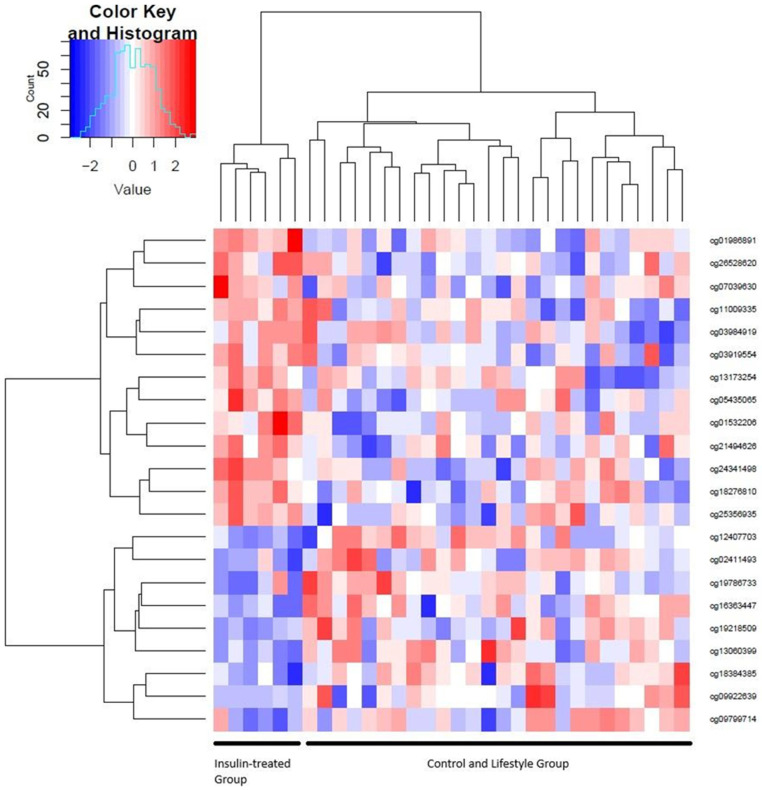
Heatmap of the 22 CpGs at antepartum visit (T1).

Additionally, we performed unsupervised hierarchical clustering to identify patient clusters based on the DNA methylation levels of these 22 CpG sites. This analysis showed two clusters. Cluster 1 contained, exclusively, the six patients from the insulin group, whereas Cluster 2 consisted of pregnant women from both the control and the lifestyle groups (Figure 1). The clustering analysis was able to split off the pregnant women with an insulin requirement from the rest. These differences in the profile of DNA methylation in the insulin group are shown in the heatmap, demonstrating a different pattern in the pregnant women with insulin versus the control and lifestyle groups (Figure 1).

### 2.3. Association of Vitamin D Levels with DNA Methylation in Genes Related to Vitamin D Metabolism

**Figure 2:**
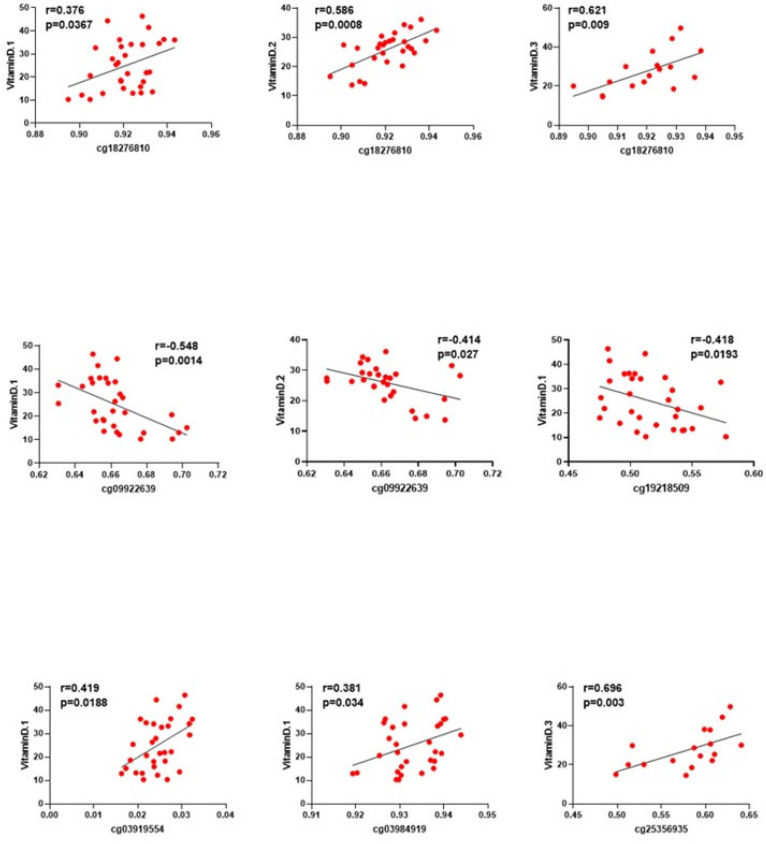
Correlations of CpG values at the antepartum visit (T1) with vitamin D levels during pregnancy. Vitamin D.1: vitamin D levels at the antepartum visit; Vitamin D.2: vitamin D levels at the postpartum visit; Vitamin D.3: vitamin D levels at one year after birth. Black line indicates the trend of the relationship. The subjects are represented by red dots.

To test if these differences in the DNA methylation levels could be associated with the vitamin D levels observed at the end of pregnancy, we performed correlation analysis between these variables at the antenatal visit. Further, due to the close relationship between vitamin D and insulin resistance, we also analyzed the association with insulin-related parameters. Of the 22 DMPs, 5 CpGs showed a statistically significant association with the vitamin D levels before birth; cg18276810 (CTNNB1), cg03919554 (FGFR3) and cg03984919 (NCOA1) were positively correlated, whereas cg19218509 (ASIP) and cg09922639 (SMAD3) showed a negative relationship (Figure 2). Furthermore, the methylation of cg18276810 (CTNNB1), cg09922639 (SMAD3), cg03984919 (NCOA1), and cg25356935 (PDZD3) was also associated with vitamin D levels after birth, at the antepartum visit (r = 0.586, *p* = 0.0008; r = 0.414, *p* = 0.027; r = 0.411, *p* = 0.026, and r = 0.497, *p* = 0.006, respectively). Finally, cg18276810 (CTNNB1) and cg25356935 (PDZD3) were positively associated even with the vitamin D levels at one year after birth, showing a strong association with vitamin D at the different points and with the same trend (Figure 2).

Regarding insulin-related parameters, we also observed a relationship between several CpGs and HOMA-IR and c-peptide levels. cg09922639 and cg03984919 at the postpartum visit were associated not only with vitamin D levels but also with insulin resistance measured by HOMA-IR after delivery (r = −0.364, *p* = 0.041 and r = 0.508, *p* = 0.003, respectively). 

## 3. Discussion

In our study’s population of pregnant women, we identified some CpG sites, in genes related to the vitamin D metabolism, that were differentially methylated at the end of pregnancy in the women with GDM who were treated with insulin versus the lifestyle group and the control group but were similar at baseline, indicating a change during pregnancy. Furthermore, some of these CpG sites were correlated to vitamin D levels at antepartum, postpartum, and one year after birth. 

In a previous study utilizing our study population (EpiDG cohort), we found that women with GDM requiring insulin had higher levels of vitamin D in comparison with those not requiring insulin and healthy controls at postpartum and 1 year after birth [8]. We suggested that epigenetic mechanisms could explain these results and proposed that insulin therapy could be favoring higher levels of vitamin D through epigenetic mechanisms in pregnant women with GDM. These results tie in with the literature on the effects of diabetes drugs on the epigenome. Garcia-Calzón et al. [12] found, in *in vitro* cells, that subjects who just received metformin had lower methylation levels of the genes SLC22A1, SLC22A3, and SLC47A1, in comparison to subjects who received insulin plus metformin or non-diabetic medication. These genes are part of the SLC superfamily and encode the three metformin transporters (OCT1, OCT3, and MATE1) in the human liver. In the in vivo cells, the methylation of SLC22A1 was lower in cells exposed to metformin and higher after insulin treatment. These results support that metformin therapy is associated with the lower DNA methylation of metformin transporter genes in the liver, suggesting that epigenetics could be a potential mechanism for metformin action. Another recent study observed that metformin improved the endometrial function of patients with Polycystic Ovarian Syndrome (PCOS) by modulating the DNA methylation of the gene promoter of HOXA10 and the expression of genes implicated in insulin signaling such as SLC2A4 and endometrial receptivity such as GAB1 [16]. In our study, DNA methylation levels of the cg16363447 (SLC34A2) were significantly lower in the insulin group versus the lifestyle group and the control group at the antepartum visit. The SLC34A2 gene is part of the SLC family, similar to those previously mentioned, so this gene could be susceptible to be influenced by drugs such as insulin and metformin. More recently, García-Calzón et al. investigated the effects of metformin on genome-wide methylation in blood in a total of 322 patients with T2DM. They related the reduction in HbA1c in patients treated with metformin, compared to controls, to the effect of this drug on different sites of PKNOX2, WDTC1, and MICB genes, with most of them being hypermethylated (88%). This supports the hypothesis that metformin increases the ratio of SAM:SAH, being that SAH is the principal methyl donor in DNA methylation [17].

In our study, the DMPs were annotated to 18 genes, and some of their CpGs were correlated with vitamin D levels at different times. First of all, vitamin D active or 1,25-dihydroxyvitamin D3 (1,25[OH]2D3) is produced by two hydroxylation reactions that occur in the liver and kidney. One enzyme that is an important participant in the process is 25-hydroxylase, which is produced by CYP2R1. CYP2R1 is part of the cytochrome P450 family, as well as CYP24A1 and CYP27B1 that are implicated in the anabolism and catabolism of vitamin D. We found cg26528620 (CYP2R1) to be differentially methylated in the insulin group versus the lifestyle group and the control group. It is reported that mutations of CYP2R1 are related to different forms of vitamin D and serum levels of vitamin D [18], so focusing on our results, insulin therapy could be modifying the DNA methylation of CYP2R1 and, as a consequence, levels of vitamin D are higher in the insulin group versus the lifestyle group and the control group.

The activities of vitamin D depend on its union with the VDR and its bind as part of a heterodimer VDR-RXR and VDR-RXR-DNA complex. Deregulation of VDR function may lead to several diseases such as diabetes. RXRA, which is part of the RXR (retinoic X receptors) family, encodes the RXRα receptor which forms a complex with the VDR. We found significant differences in cg24341498 (RXRA) between the insulin group versus the lifestyle group and the control group.

Furthermore, NcoAs are the major coactivators of the VDR. NcoA1 binds to the liganded VDR-RXR-DNA complex and stabilizes VDR and RXR [19]. In our results, cg01986891 (NcoA3), cg12407703 (NcoA1), and cg03984919 (NcoA1) were differentially methylated between the insulin group versus the lifestyle group and the control group, and focusing on this, we could suggest a different regulation of the VDR-RXR complex through the epigenetic mechanism in patients treated with insulin versus the lifestyle group and the control group. Additionally, methylation levels of cg03984919 (NcoA1) were positively correlated with vitamin D levels at the antepartum visit, suggesting that this correlation is related to VDR-RXR complex regulation.

Furthermore, it was validated by Yu et al. through a chromatin immunoprecipitation assay that SMAD3 can bind to the VDR promoter and the VDR can bind to the SMAD3 promoter. This reciprocal activation between SMAD3 and VDR transcription factors defines the vitamin D-mediated oxidative stress to prevent cerebral ischaemia–reperfusion injury [20]. Also, the VDR/TFGβ/SMAD3 signaling cascade could have an effect on fibrosis and other liver-related events [21]. We found that methylation levels of cg09922639, cg21494626 (SMAD3), and cg13173254 (VDR) are statistically different in the insulin group versus the lifestyle group and the control group. We also found a negative relationship between cg09922639 (SMAD3) and vitamin D levels at the antepartum visit. These differences could be influenced by the DNA methylation due to insulin treatment in the VDR/TFGβ/SMAD3 complex.

Regarding FGF23, it acts in the kidney and suppresses the transcription of the enzyme 1-α-hydroxylase and activates the transcription of 24-hydroxylase via an αKlotho-dependent signaling mechanism involving FGFR1, FGFR3, and FGFR4 [22].

We found differences in DNA methylation at cg03919554 (FGFR3) and cg07039630 (FGFR1) between groups and, in addition, a positive relationship between cg03919554 (FGFR3) and vitamin D levels at the antepartum visit was shown.

The main limitation of our study is the sample size; however, this point is counteracted by conducting a hypothesis-driven investigation based on our previous results. Additionally, we do not have RNA samples to quantify the effect of DNA methylation on gene expression, and consequently on vitamin D levels.

## 4. Materials and Methods

### 4.1. Population Study

The participants in this study are part of the project “Effect of gestational diabetes on the epigenome of the mother and the offspring during the first 1000 days. Identification of potential epigenetic biomarkers of risk of T2DM and obesity” (EPIDG). Characteristics of the whole cohort have been published previously [23]. Pregnant women attending the Diabetes and Pregnancy Unit of the Hospital Universitario Virgen de la Victoria, following a positive O’Sullivan test, were eligible for recruitment. National Diabetes Data Group (NDDG) criteria were followed to diagnose GDM [24]. First, a screening test was performed in pregnant women between 24 and 29 weeks of pregnancy, with an oral glucose tolerance test (OGTT) of 50 g, in primary centers. Then, an OGTT of 100 g was performed in those women with a positive screening test (>7.7 mmol/L). Patients were diagnosed with GDM if glucose values were above the threshold at at least two points: fasting >5.8 mmol/L; after 2 h > 9.2 mmol/L; after 3 h > 8.0 mmol/L. Pregnant women with a normal OGTT-100 were considered controls (non-GDM). Informed consent was obtained from all subjects. The study was approved by the Institutional Review Board at the Hospital Universitario Virgen de la Victoria de Málaga, Spain.

### 4.2. Samples Extraction, DNA Isolation, and Bisulfite Conversion

Peripheral blood samples were collected at four different time points: T0, during the diagnostic visit (24–29 weeks); T1, at the antepartum visit (37–38 weeks); T2, at the postpartum visit (8–10 weeks postpartum); and at T3 (a year after childbirth). Blood and serum were stored at −80 °C. Clinical, anthropometric, and biochemical variables were collected during each visit. 

For DNA methylation measurement, a subgroup of 32 samples was selected, 16 of which where from participants with GDM and 16 of which were from participants who did not have GDM. From the GDM group, 6 required insulin treatment, and 10 received recommendations about lifestyle changes. Samples from the control group were matched by age, gestational age, and pre-pregnant BMI to avoid cofounding factors in the methylation data analysis.

Peripheral blood DNA was isolated from samples at T0 and T1 using the Qiamp DNA Blood Mini Kit (Qiagen, Hilden, Germany) according to the manufacturer’s instructions. Quality and concentration of DNA was measured using a Qubit 3.0 Fluorometer with a Qubit dsDNA HS Assay Kit Fluorometer (Thermo Fisher Scientific, Waltham, MA, USA). A total of 500 ng of genomic DNA was bisulfite-treated with the Epitect Bisulfite Kit (Qiagen, Germany) for posterior DNA methylation analysis.

### 4.3. DNA Methylation Analysis

An Epigenome-wide association study (EWAS) was previously performed using the Methylation EPIC Bead Chip Kit (Illumina, San Diego, CA, USA) in the 32 patients (16 with GDM and 16 without GDM) at two different times, T0 and T1 [23]. Raw data were processed using R package ChAMP version 2.9.10 [25]. Filtering probes was performed in probes with a detection *p*-value above 0.01 in one or more samples, probes with a beadcount less than 3 in at least 5% of samples, non-CpG probes, probes with SNPs, probes that align to multiple locations, and probes on the X or Y chromosome. Intra-cell-type normalization was carried out using the beta-mixture quantile Normalization (BMIQ) method. To correct for the differences in methylation resulting from differences in cellular heterogeneity, Houseman correction was used [26]. Beta values were calculated to obtain the methylation levels.

### 4.4. Vitamin D Gene Selection and CpGs Extraction

**Figure 3:**
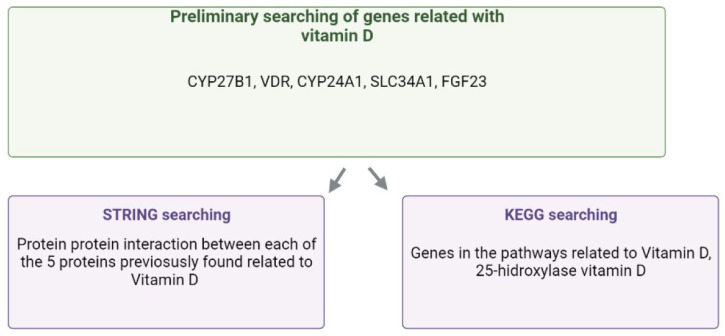
Workflow selection of genes related to vitamin D.

A preliminary bibliographic search was performed to identify which genes were directly related to vitamin D. Also, we looked for those genes that could interact with the genes directly related to vitamin D. Therefore, the Kyoto Encyclopedia of Genes and Genomes (KEGG) [27] and the String platform [28] were used to look for those genes. In the KEGG, search criteria were “vitamin D” and “vitamin D 25-hydroxylase”. In STRING, a Protein–Protein Interaction (PPI) was searched for each gene directly related to vitamin D (Figure 3). 

CpGs’ annotations were obtained using the IlluminaHumanMethylationEPICanno.ilmn12.hg19 package from R software 4.0.4 [29]. Then, M-values and Beta-values were extracted from all CpGs in the genes related to the vitamin D pathway.

### 4.5. Statistical

R studio software version (4.0.4) was used for all statistical analyses (https://www.r-project.org (accessed on 1 August 2024)). Due the small samples, non-parametric tests were performed to detect the differences between the three groups: those without GDM, those with insulin-treated GDM, and those with GDM who received lifestyle change recommendations. First of all, a Kruskal–Wallis was performed at T1 with the aim to test which CpG sites were differentially methylated (DMPs) (*p* < 0.05) at the end of pregnancy in the insulin-treated group compared with the other two groups. A post hoc analysis with a Mann–Whitney U-test was performed to identify which groups had differences between them. Finally, we tested which DMPs at T1 were not different at T0 by Kruskal–Wallis, and CpGs with a threshold of *p* > 0.04 were selected. The D3heatmap and ggplots packages in R were used to perform unsupervised hierarchical clustering to identify patient clusters based on DNA methylation levels of the DMPs at T1 and those that were equal at T0. 

Finally, correlations were performed using Spearman’s test with vitamin D levels at different time points (T0, T1, T2, and T3). The correlation matrix was obtained with corrplot and GGally packages in RStudio, and the threshold was *p* < 0.05.

## 5. Conclusions

In conclusion, in our study’s population of pregnant women, we found that DNA methylation in genes related to vitamin D metabolism differs in the last trimester of pregnancy between women with GDM who were treated with insulin versus diet-treated women and controls, supporting our previously suggested hypothesis that the higher levels of vitamin D observed in women with GDM exposed to insulin, in comparison to women with GDM who were not treated with insulin and controls, could be, at least in part, mediated by epigenetic mechanisms.

## Figures and Tables

**Table 1:** Characteristics of the participants and a comparison between groups.

	Control	Lifestyle	Insulin-Treated	*p* Value
Baseline (n)	16	10	6	
Age (years)	34.1 ± 4.5	31.6 ± 2.7	37.5 ± 3.4	**0.018**
BMI before pregnancy (kg/m^2^)	25.5 ± 4.1	23.8 ± 2.9	28.4 ± 4.6	0.113
Fasting glycemia (mg/dL)	4.5 ± 0.4	4.6 ± 0.3	5.5 ± 0.4	**0.002**
HbA1c (%)	5.1 ± 0.3	5.1 ± 0.4	5.5 ± 0.3	**0.03**
25-OH-D (ng/mL)	24.8 ± 9.2	21.3 ± 8.2	30 ± 4.6	0.115
Vitamin D				0.503
supplements (%)				
-No	81.3	80.0	100.0	
-Yes	18.8	20.0	0.0	
HOMA-IR	1.6 ± 0.7	1.3 ± 0.5	3.3 ± 0.9	**0.002**
MD adherence score	7.7 ± 1.3	7.2 ± 2.2	6.8 ± 1.7	0.303
Daily walking/cycling (min)	65.8 ± 47.3	54.9 ± 59.8	23.9 ± 27.7	0.108
Antepartum visit (n)	16	10	6	
Weight gain in pregnancy (kg)	11.0 ± 4.1	8.2 ±4.7	11.1 ± 4.9	**0.26**
Fasting glycemia (mg/dL)	4.3 ± 0.8	4.3 ± 0.9	4.6 ± 0.9	0.786
HbA1c (%)	5.2 ± 0.3	5.3 ± 0.3	5.6 ± 0.3	**0.045**
25-OH-D (ng/mL)	23.3 ± 8.6	21.3 ± 10.9	35.4 ± 9.6	**0.028**
HOMA-IR	3.5 ± 7.3	1.7 ± 1.0	7.2 ± 5.4	**0.016**
Postpartum visit (n)	16	8	6	
BMI (kg/m^2^)	27.1 ± 4.1	23.8 ± 2.0	28.9 ± 3.5	**0.027**
Fasting glycemia (mg/dL)	4.5 ± 0.3	4.4 ± 0.5	4.9 ± 0.4	0.07
HbA1c (%)	5.4 ± 0.3	5.4 ± 0.4	5.5 ± 0.3	0.874
25-OH-D (ng/mL)	25.0 ± 6.4	23.8 ± 3.8	31.6 ± 2.5	**0.008**
HOMA-IR	1.2 ± 0.7	0.99 ± 0.8	1.8 ± 0.6	0.07
1 year after birth visit (n)	7	5	5	
BMI (kg/m^2^)	26.4 ± 4.2	26.1 ± 6.5	31.1 ± 6.0	0.19
Fasting glycemia (mg/dL)	4.8 ± 0.5	5.1 ± 0.5	5.1 ± 0.5	0.751
HbA1c (%)	5.3 ± 0.1	5.4 ± 0.4	5.5 ± 0.4	0.670
25-OH-D (ng/mL)	25.1 ± 7.0	22.8 ± 6.8	36.3 ± 12.2	0.120
HOMA-IR	1.3 ± 0-8	1.9 ± 0.8	2.4 ± 1.2	0.894
MD adherence score	8.0 ± 1.4	7.6 ± 2.3	6.6 ± 1.5	0.378
Daily walking/cycling (min)	32.7 ± 24.6	33.58 ± 23.4	31.4 ± 27.2	0.539

25-OH-D: 25-hydroxyvitamin D; HOMA-IR: homeostasis model assessment of insulin resistance index; MD: Mediterranean diet; min: minutes.

**Table 2:** CpG sites statistically different at the antepartum visit and equal at the diagnostic visit.

CpG_lD	Gene	Island Position	Gene Position	*p*.Value T0	*p*.Value TI
cg19218509	ASP	Island	Body	0.109	0.014
cg18384385	CAI	OpenSea		0.048	0.020
cg01532206	CA7	Island		0.054	0.029
cg182.76810	CTNNBI	N Shore		0.135	0.015
cg26528620	CYP2R1	Island	Bod	0.657	0.017
cg07039630	FGFRI	OpenSea		0.623	0.033
cg03919554	FGFR3	Island		0.487	0.045
cg13060399	GALNT3	OpenSea	Body	0.227	0.015
cg11009335	GALNT3	S Shore	TSS1500	0.128	0.042
cg19786733	KLB	S Shore	Body	0.104	0.034
cg02411493	NADSYNI	O ensea	Bod	0.488	0.032
cg12407703	NCOAI	OpenSea		0.926	0.013
cg03984919	NCOAI	OpenSea		0.547	0.040
cg01986891	NCOA3	OpenSea		0.065	0.003
cg09799714	PDZD3	OpenSea		0.956	0.032
cg25356935	PDZD3	OpenSea		0.061	0.007
cg05435065	POR	Island	TSS1500	0.102	0.013
cg24341498	RXRA	Island	TSS1500	0.094	0.014
cg16363447	SLC34A2	Island	Bod	0.987	0.012
cg09922639	SMAD3	OpenSea		0.606	0.022
cg21494626	SMAD3	OpenSea	Body	0.267	0.010
cg13173254	VDR	OpenSea		0.046	0.039

*p*.value calculated with Kruskal–Wallis comparison, T0 at the diagnostic visit, T1 the antepartum visit.

## Data Availability

The data sets from EPIDG used during the current study are available from the corresponding author on reasonable request.

## Supplementary Materials

The following supporting information can be downloaded at: https://www.mdpi.com/article/10.3390/ijms251910576/s1.
