# Supplementary material for: Differences in DNA Methylation in Genes Involved in Vitamin D Metabolism Are Related to Insulin Requirement in Pregnant Women with Gestational Diabetes Mellitus
Source: Int J Mol Sci. 2024 Oct 1;25(19):10576. doi: 10.3390/ijms251910576 (PMC11476386; doi:10.3390/ijms251910576)
Supplement: Supplementary file 1 [file ijms-25-10576-s001.zip › ijms-3216260-supplementary.pdf]

Supplementary Table S1. Gene list related with Vitamin D

Gene list related with vitamin D
CYP2R1
GC
PTH
FDX1
KL
DHCR7
SLC34A3
CA5B
CA13
CA5A
CA7
CA14
CA1
CA8
NCOA1
RXRA
SMAD3
MED1
CTNNB1
BAZ1B
EP300
NCOA3
PDZK1
PDZD3
SLC34A2
PFN3
SLC20A2
SLC17A1
FGFR1
FGFR4
FGFR2
FGFR3
GALNT3
KLB
MAPK3
KAL1
PHEX
DFCR7
NADSYN1
POR
ASIP
IRF4

**CYP27B1**
**VDR**
**CYP24A1**
**SLC34A1**
**FGF23**
